# Investigation of a special neutralizing epitope of HEV E2s

**DOI:** 10.1007/s13238-014-0115-3

**Published:** 2014-11-22

**Authors:** Min You, Lu Xin, Yi Yang, Xiao Zhang, Yingwei Chen, Hai Yu, Shaowei Li, Jun Zhang, Zhiqiang An, Wenxin Luo, Ningshao Xia

**Affiliations:** 1 National institute of diagnostics and vaccine development in infectious diseases, Xiamen University, Xiamen, 361105 China; 2 Texes Therapeutics Institute, The Brown Foundation of Molecular Medicine, University of Texas Health Science Center at Houston, Houston, TX77030 USA; 3 Potomac Affinity Proteins, 9601 Medical Center Drive, Suite #330, Rockville, MD 20850 USA

**Dear Editor**

After 14-years of development, the first prophylactic vaccine against the Hepatitis E virus (HEV) has been marketed since 2012 (Wu et al., 2012). However, the neutralizing epitopes of HEV are not completely defined. E2s, a protruding homodimer domain of HEV capsid protein, is responsible for interacting with host cells to initiate infection (Li et al., 2009; Li et al., 2005). It was shown that two monoclonal antibodies (mAb), 8C11 and 8H3, could neutralize the infectivity of HEV in rhesus, and the two mAbs bind to different neutralizing conformational epitopes on E2s (Zhang et al., 2005). The epitope for 8C11 was reported based on the crystal structure of HEV protruding domain E2s in complex with 8C11 Fab (Tang et al., 2011). In a HEV antibody panel consisting of 30 mAbs, including 8C11, none of them can cross block the binding of 8H3 to E2s. These findings suggest that 8H3 recognizes a special epitope which is different from the epitope for 8C11 and other mAbs. X-ray crystallography is not a viable option to identify the 8H3 epitope because 8H3 binds to the E2s with a low affinity (Zhang et al., 2005).

Conformational epitopes, which often escape identification by linear peptide screening, can be identified and characterized from studies with mimotopes (Cardoso et al., 2009). Most mimotopes obtained from phage displayed peptide libraries can be employed to facilitate the identification of novel peptide sequences that mimic binding sites for mAbs (Mayrose et al., 2007). We panned three phage-displayed peptide libraries (ph.D.-C7C and ph.D.-12 displaying peptide on the pIII protein, and lib C10C displaying peptide on the pVIII protein) to select 8H3 mimotopes (Table S1). After three rounds of panning, phage clones were tested for binding specificity to 8H3. Mimotopes which reacted to 8H3 without cross-reacting with three HEV related antibodies (8C11, 12G8, and 8G12) and two HEV non-related antibodies (13D4 against AIV and 42B6 against HBV) were considered positive. Finally, 21 mimotopes to 8H3 were obtained for further analysis (Table S2).

The 21 mimotopes to the 8H3 mAb were processed individually by three efficacious prediction programs, Pep-3D-Search program, Pepsurf and EpiSearch for 8H3 epitope mapping (Huang et al., 2008; Mayrose et al., 2007; Negi and Braun, 2009). The E2s structure of the HEV (PDB: 3GGQ) was used as a template for epitope prediction (Li et al., 2009). The mimotope sequences listed in Table S2 were used to deduce the best cluster by default parameters. The prediction results from the three programs were shown in Table S3. Overlapping regions of the predicted clusters from the three programs, composed of Gln^482^-Thr^484^, Ser^487^-Gly^490^, Ser^527^-Pro^540^, Tyr^559^-Asn^560^, Asn^562^-Gln^568^, Asn^573^ and Ser^582^-Thr^586^, were considered parts of the 8H3 epitope. These overlapping regions (shown in rose red in Fig. 1A) are located on the groove of E2s, and they are independent from the epitope of 8C11.

**Figure 1:**
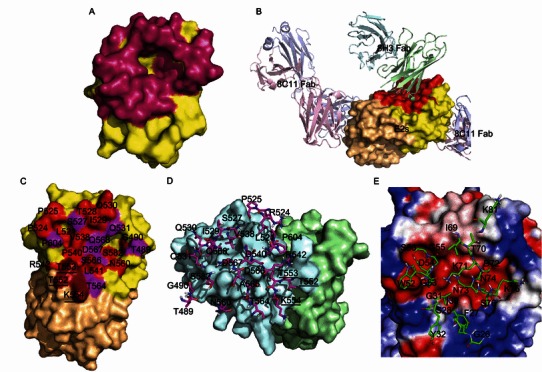
**The predicted binding-region of 8H3-E2s**. (A) The sites in rose red are the overlapping epitope regions from the prediction results of three programs, Pepsurf, Pep-3D-Search, and Epi-search. (B) The predicted binding-region (in red) of 8H3-E2s (E2s, PDB: 3KRD) by ZDOCK. (C) Surface representations of E2s highlighted the interacting epitope residues in dark shade. The epitope residues shown in purple correspond to their interactions to 8H3 by hydrogen bonds. The epitope residues shown in brown are located on the second subunit (in orange) of E2s. (D) The major clusters of 8H3 epitope on E2s are shown in ball and stick model and colored in deep purple. The epitope residues labeled with underline are located on the second subunit of E2s. The 8H3 Fab is shown as surface representation, H-chain is shown in light blue, and L-chain is green. (E) Depicts the electrostatic potential surface of the epitope on the E2s (red, negative; blue, positive; and gray, neutral) with the key residues for interaction from 8H3 Fab represented as sticks. The figure was prepared using the program PyMOL (Delano, 2002)

The prediction results based on the mimotope sequences provide general information on the binding region on the antigen, but it does not provide the details on antigen-antibody contacts, for example, the amino acids involving in hydrogen-bonding contacts and the binding sites on the antibody. The rigid-body protein-protein docking program ZDOCK was then used to map the antigen-antibody contact sites. Fast Fourier Transform (FFT) algorithm was applied to perform a global docking to search for potential binding positions of two component proteins (Pierce et al., 2014). Since validity of the ZDOCK analysis is affected by the accuracy of the search algorithm as well as the protein-protein complex to be predicted, some of the top-scoring predictions resulted from the soft scoring function of the program could be false positives (Wiehe et al., 2008). Combining the results from epitope prediction softwares based on mimotope and ZDOCK may lead to a more reliable result. The overlapping regions (Gln^482^-Thr^484^, Ser^487^-Gly^490^, Ser^527^-Pro^540^, Tyr^559^-Asn^560^, Asn^562^-Gln^568^, Asn^573^ and Ser^582^-Thr^586^) predicted from the three programs were further investigated by ZDOCK. The 3-dimensional model of mAb 8H3 was generated by a homology modeling protocol. Given the facts that the epitope of antibody 8H3 is different from that of 8C11, and the binding of 8H3 to E2s can be enhanced by 8C11 (Zhang et al., 2005), the structure of 8C11 Fab-E2s complex (PDB: 3RKD) was used as the antigen for the ZDOCK program to search for the best combination model. As a result, the surface on antigen E2s for binding to antibody 8H3 were shown in red (Fig. 1B). The regions of E2s binding to 8H3 were further analyzed and shown in dark shade (Fig. 1C). In addition to the same three regions (Ser^527^-Thr^535^, Tyr^559^-Asn^560^, and Tyr^584^-Thr^586^) predicted by the three programs based on mimotopes, there are four additional binding regions (Leu^521^, Arg^524^-Pro^525^, Leu^570^, Ala^602^-Pro^604^) on the first subunit and one region (Thr^552^-Lys^554^) on the second subunit from the prediction by ZDOCK (Fig. 1C and 1D). All the predicted interaction regions are located on the groove or near-groove of the E2s dimer structure.

In the predicted model from ZDOCK, the specificity of 8H3 Fab to E2s is dictated by seven major interactions at Thr^489^-Gly^490^, Arg^524^-Gln^531^, Pro^540^, Asn^560^, Thr^564^-Gln^568^, S^582^, and Ala^602^-Pro^604^ of E2s (Fig. 1C and 1D). There are six hydrogen-bonding contacts from Thr^489^, Ser^527^, Gln^531^, Thr^564^, Gln^568^ and Pro^604^ of E2s to 8H3 Fab (Fig. 1C and 1D). Three hydrophobic loop regions of 8H3 heavy chain ^H26^GFS^H28^, ^H52^WGDGS^H56^, and ^H71^KDN^H73^ are in close contact with E2s (Fig. 1E).

To verify the prediction results functionally, all predicted sites on E2s with solvent accessibility were individually mutated to Ala and the mutants were expressed in *E. coli*. The wild type E2s and its mutants were subjected to non-reducing SDS-PAGE and Western blotting against 8H3. All samples except Thr564Ala and Ser582Ala were resolved mainly as dimers (Fig. 2A). In previous studies, we have demonstrated that the dimerization of E2s is essential for HEV-host interactions and antibody neutralization, and Thr^564^ and Ser^582^ can stabilize the dimer (Li et al., 2009; Li et al., 2005). Therefore, the Thr564Ala and Ser582Ala mutants lose the binding ability to 8H3 due to the collapse of dimeric form of E2s. The Western blotting results showed that the wild type dimeric E2s and 15 mutants, including the ones with mutated sites (Thr^489^, Ser^527^, Gln^531^, Gln^568^ and Pro^604^) responsible for binding to 8H3 by hydrogen-bond as the prediction results of ZDOCK, were reactive with 8H3 (Fig. 2A). Since Ile529Ala and Asn560Ala mutants in dimeric form abolished the 8H3 reactivity, Ile^529^ and Asn^560^ contribute to the binding of 8H3 with E2s (Fig. 2A).

**Figure 2:**
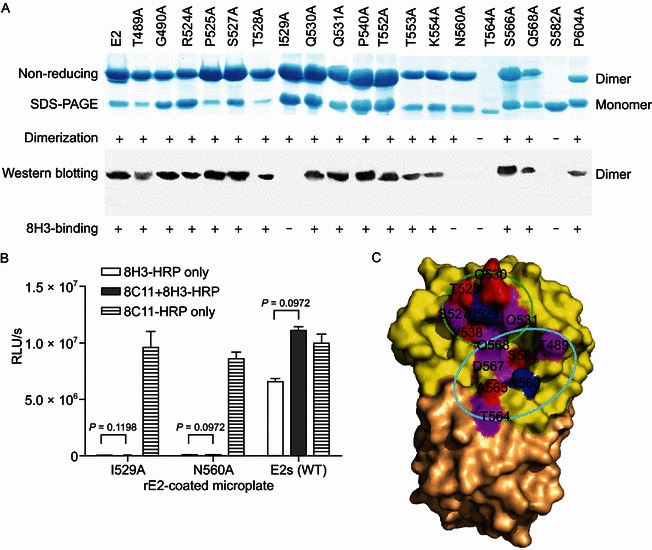
**Functional validation of the predicted epitope of 8H3**. (A) Mutational studies on the predicted binding-region of 8H3. All residues from the predicted binding-region were mutated to Ala. The wild type E2s and its mutants were subjected to non-reducing SDS-PAGE and Western blotting with 8H3. [+] denotes dimerization or reactivity with 8H3, [−] denotes loss of dimerization or reactivity with 8H3. (B) Effects of 8C11 on the binding of 8H3 with E2s mutants. (C) Residues around the two key amino acid residues in dark blue Ile^529^ and Asn^560^ in 6 Å are circled in light green and light blue, respectively. The sites in red and purple are the predicted epitope of 8H3 and the residues with interactions to 8H3 by hydrogen bonds are shown in purple

We have shown previously that mAb 8C11 enhances the binding of 8H3 to E2s (Zhang et al., 2005). If 8C11 can’t enhance the binding of 8H3 to the E2s mutants, the E2s mutants can be assumed of losing the binding capacity to 8H3 and the epitope of 8H3 on E2s was destroyed by the site mutations. In order to investigate the enhancement of 8C11 on the binding of 8H3 to the Ile529Ala or Asn560Ala mutants, CLIA (chemiluminescent immunoassay) was carried out using microplates coated with purified E2s mutants and pre-incubated with buffer containing 8C11, then reacted with HRP labeled 8H3. Binding of the labeled antibodies was measured in RLU (relative light unit). The results showed that the Ile529Ala and Asn560Ala mutants retained the binding activity to 8C11, but they can’t bind to 8H3 with the presence of 8C11 (Fig. 2B), suggesting that the epitope of 8C11 was retained, but the epitope of 8H3 was damaged in mutants Ile529Ala and Asn560Ala. From the structure of E2s (PDB: 3GGQ or 3RKD), we found that the amino acid residues around Ile^529^ in 6 Å included Gln^530^, Thr^528^, Gln^531^, Ser^527^, Val^538^ and Gln^568^, and residues around Asn^560^ in 6 Å included Thr^489^, Ser^582^, Asp^567^, Ala^565^ and Thr^564^ (Fig. 2C). The mutations of Asn560Ala and Ile529Ala may have resulted in conformational changes of the 8H3 epitope, especially those amino acid residues located around Asn^560^ and Ile^529^ in 6 Å. These results collectively indicated that Ile^529^ and Asn^560^ were the key sites to maintain the epitope of 8H3 and confirmed the prediction results that 8H3 targets the groove region of E2s.

In summary, we predicted the epitope of 8H3 on E2s by epitope prediction softwares based on the combined approaches of mimotopes and ZDOCK. The antibody 8H3 recognizes the epitope composed of Thr^489^-Gly^490^, Arg^524^- Pro^525^, Ser^527^-Gln^531^, Pro^540^, Asn^560^, Thr^564^-Gln^568^, Ser^582^, and Ala^602^-Pro^604^ on the groove regions of the HEV E2s. Mutation analysis demonstrated that Ile^529^ and Asn^560^ on E2s were the most crucial residues for its interaction with 8H3, and they were the key sites to maintain the epitope of 8H3. Our data presented here reveals for the first time a special nondominant neutralizing epitope located on the groove region of E2s. Because 8H3 binds to E2s region of HEV which has been proposed to mediate the first contact with host cells to initiate viral infection (He et al., 2008; Li et al., 2009; Tang et al., 2011), our results may have broad implications in understanding the protection mechanism of HEV vaccines and drugs designed against the HEV virus. Additionally, this is the first report of epitope mapping by combining the prediction results from mimotopes and ZDOCK. This approach may be particularly effective in investigations of special epitopes.

## Electronic supplementary material

Below is the link to the electronic supplementary material.

Supplementary material 1 (PDF 131 kb)
