# Supplementary material for: Investigation of a special neutralizing epitope of HEV E2s
Source: Protein Cell. 2014 Nov 22;5(12):950–3. doi: 10.1007/s13238-014-0115-3 (PMC4259887; doi:10.1007/s13238-014-0115-3)
Supplement: Supplementary file 1 — Supplementary material 1 (PDF 131 kb) [file 13238_2014_115_MOESM1_ESM.pdf]

SUPPLEMENTARY MATERIALS

Investigation of a special neutralizing epitope of HEV E2s

Min You[1], Lu Xin[1],Yi Yang[1], Xiao Zhang[1], Yingwei Chen[3], Hai Yu[1],

Shaowei Li[1], Jun Zhang[1], Zhiqiang An[1,2], Wenxin Luo[1,*], Ningshao Xia[1]

[1] National institute of diagnostics and vaccine development in infectious diseases,

Xiamen University, Xiamen 361102 , China;

[2]Texes Therapeutics Institute, the Brown Foundation of Molecular Medicine,

University of Texas Health Science Center at Houston, Houston, TX77030;

[3] Potomac Affinity Proteins, 9601 Medical Center Drive, Suite #330, Rockville, MD

20850,USA.

Corresponding author: Wenxin Luo, at the National Institute of Diagnostics and Vaccine Development in infectious diseases, Xiamen University, Xiamen 361102, China; Tel: 0592-2188657; Fax: 0592-2181258; E-mail: *wxluo@xmu.edu.cn*

Running title: A special neutralizing epitope of HEV

KEYWORDS: neutralizing epitope, Hepatitis E virus (HEV), mimotope, ZDOCK

Supplementary Tables

Table S1. Phage display libraries used in 8H3 mimotope selection.

Library	Fusion protein	Random peptide	Complexity
ph.D.TM-C7C	pIII	cyclic	1.2×10^9
ph.D.TM-12	pIII	linear	2.7×10^9
lib C10C	pVIII	cyclic	2.7×10^9

Table S2. Mimiotpes selected with 8H3 mAbs

Mimotopes of 8H3		
ph.D.TM-12	lib C10C	ph.D.TM-C7C
YTTKLIPPWLST	LEVSRWAWYN	QGILDYY
GINPQLLKVPTP	QWLDYWDRQK	TSPALPW
TPDNTYRTYMQG	EMRRDAWFGA	NGSLDYY
AQNPLWYLDSST	YWLYMVLGEV	YEPSLML
TPKMTNYLDYMY	ELNRREYYGS	VYPSRMM
TTQTHSNAWKLF		APWDHAS
TPSELNRWQFYF		SLPSLMM
ELNGWRLMGQSA		
YYTDYNLESYQS		

Table S3. Possible epitope regions predicted by bioinformatic

EpiSearch			Pep-3D search			Pepsurf		
Lin 7	Lin 12	C 10	Lin 7	Lin 12	C 10	Lin 7	Lin 12	C10
Score: 6.09 [0.870]	Score: 8.04 [0.893]	Score: 4.10 [0.820]	Cluster 1	Cluster 1	Cluster 1	Cluster 1 （26.122）	Cluster 1 （417.73）	Cluster 1 （25.171）
463, 464, 466, 467, 469 ~ 527, 540, 602	480 ~ 482, 485, 487, 489, 490, 492, 494, 543, 544, 556, 557, 559 ~ 563, 566 ~568, 570, 582, 584, 585, 587	479, 481, 485, 492, 492, 494, 496, 497, 512, 570, 574 ~ 576, 578	472, 480 ~ 482, 485, 486, 490, 493, 494, 527, 529, 531, 540 ~ 545, 548, 554, 557, 558, 560, 562 ~ 570, 580 ~ 582, 586, 588 ~ 590, 592, 601, 602	482, 486, 491 ~ 492, 517 ~ 519, 528 ~ 534, 537, 540, 541, 543, 558, 560 ~ 563, 567 ~ 569, 573, 581, 583, 600, 602	470, 472, 474, 481, 483, 484, 486, 492, 493, 502, 515, 516, 530 ~ 534, 536, 537, 547 ~ 557, 560, 561, 570, 572 , 573, 579 ~ 581, 581, 588, 595, 597	459 ~ 461, 463, 464, 466, 505, 510, 514, 521, 524 ~ 527	479 ~ 486, 492, 494, 507 ~ 510, 512 ~ 514, 528 ~ 530, 532, 534 , 535, 538, 540, 568, 570, 576, 578, 590	460, 461, 463, 464, 466, 469, 470, 473, 474, 501, 503, 506 ~ 508, 548, 549, 552 ~ 555, 596, 598
Score: 5.13 [0.733]	Score: 7.57 [0.841]	Score: 3.77 [0.754]	Cluster 2			Cluster 2 （13.074）	Cluster 2 （400.32）	Cluster 2 （8.3771）
546, 548, 555 ~ 557, 559 ~ 562, 582, 584, 587	514, 515, 517, 518, 520, 521, 525 ~ 531, 534, 535, 537, 538, 540, 568, 570, 573, 574, 578	463, 464, 466, 467, 469, 521 ~ 524, 526, 527, 537, 538, 602	461 ~ 463, 501, 514 ~ 519, 573 ~ 577			480 ~ 489, 559, 561	544, 556 ~ 563, 565, 582, 584 ~ 587	544, 556, 557, 560 ~ 562, 587
Score: 4.90 [0.700]	Score: 7.24 [0.804]					Cluster 3 （5.8935）		Cluster 3 （6.0392）
480 ~ 485, 487 ~ 490, 492, 559, 585, 586	479 ~ 485, 487 ~ 490, 492, 494,531, 556, 559, 567, 568, 570, 578, 582, 584 ~ 586					549, 550, 555, 594		480 ~ 482, 484, 485, 492 ~ 531, 534

MATERIALS AND METHODS

Phage libraries and mouse monoclonal antibodies

The phage displayed random peptide libraries (ph.D. TM) expressing peptide fused to pIII of the filamentous phage M13 were obtained from New England Biolabs (Ipswich, Massachusetts, USA). The phage displayed random libraries, expressing cysteine-flanked peptide, disulfide bridging, fused to the pVIII of M13 was a kind gift of Prof. Jonathan Gershoni (Tel Aviv University, Israel). The libraries used are listed in Table S1. The mouse monoclonal antibody 8H3 was prepared in-house.

Bio-panning and selection of phage peptide

An aliquot of 1ul of the peptide library containing 2.9×10^{11} peptide bound phages was pre-mixed with 30μg target mAb in 200μl Tris buffer saline (TBS) with 0.1% Tween-20 for 20 min at room temperature. Then 15μl protein A blocked by 5mg/ml BSA in 0.1 M $NaHCO_3$ (pH 8.6) was added and incubated for 20min. The unbound phage particles were removed by washing with TBS containing 0.5% Tween-20 (TBST), and the bound phages were eluted with 0.2 M Glycine-HCl (pH 2.2) and immediately neutralized by 1M Tris-HCl（pH 9.1). The harvested phage was

amplified by the log-phase *E coli ER2738* and purified by precipitation with 20% PEG 8000, 2.5M NaCl. This was then incubated with mAb and screened 2 times. With each subsequent wash, at least 5 washes per experiment, the buffer was changed from 0.1% Tween-20 TBS in the 1st round of screening to 0.5% Tween-20 TBS in the 2nd round. This had the effect of enriching phage peptides possessing antibody binding specificity. After the third round, phage clones were screened for target mAb reactivity by ELISA. An HEV related antibody and 2 non-related antibodies directed against other viruses were used as negative controls. A clone with three time higher reactivity to target mAb than control was considered positive. Single-stranded phage DNA of positive clone was prepared and sequenced by BioAsia Biology Inc. (Shanghai, China).

Epitope mapping of mimotopes by 3 prediction programs

The model of E2s (PDB: 3GGQ or 3RKD) was used as a template for epitope prediction. For Pepsurf, BLOSUM62 was chosen for the substitution matrix, and the gap penalty value was set to -0.5. The Surface Racer program (Tsodikov et al., 2002) with a probe sphere of radius 1.4 Å, corresponding to the length of a water molecule

and used to calculate the solvent accessible surface area (SASA) of each atom of residues in the study antigen. The results from the Surface Racer, the Pep-3D-Search program (Huang et al., 2006) was used to define the solvent exposed residues of the study antigen and predict epitopes by mimotopes (distance parameter CA , distance threshold 7). When using the Epi-search, the Areacutoff was sett to 10.0, and Accuracy cutoff was set to 3.0.

Epitope mapping by ZDOCK

Antibody 8H3 generated by the homology modeling protocol and antigen 3RKD downloaded in the Protein Data Bank (PDB) were submitted to ZDOCK program for the best combination modes. ZDOCK performs an exhaustive rigid-body search in 6D space, which is separated into three rotational degrees of freedom. After 6° angular sampling, 2000 predictions were shown in the result viewing window. The top 26 poses in the largest clusters were reranked with a more detailed scoring function by ZRANK (Hwang et al., 2010; Vreven et al., 2013). The best pose scored by shape complementarity, electrostatics and an atomic statistical potential was then carried out for interface analyzing run to map the epitope of E2s recognized by 8H3.

Construction and expression of rE2 mutation

The site-direct mutated insert, obtained by PCR amplification and overlapping, was digested by *Nde*I and *EcoR*I and linked to the pTO-T7 vector (Luo et al., 2000). The recombinant plasmid was transformed into the *E.coli ER2566* strain. The transformant was cultured in LB medium at 25°C for 5 h and then further incubated for 4 h in the presence of 0.2mM of isopropylthio-β-D-galactoside (IPTG). The cells were lysed by sonication. Triton X-100 was added to a final concentration of 2%. The sonicate was allowed to stand at 4°C for 30 min and centrifuged at 10,000×g for 10 min. The pellet was resuspended in 4M urea buffer (200mM Tris, pH 8.5, 5mM EDTA, 100mM NaCl, 4M urea), allowed to stand for 10 min at room temperature and centrifuged at 10,000×g.

The reactivity of rE2 mutants to 8H3 mAbs

Purified rE2 mutants suspended in a loading buffer (50mM Tris pH 6.8, 2% SDS, 5% 2-mercaptoethanol, 0.01% bromophenol blue, 8% glycerol) were electrophoresed in 12% SDS-PAGE and then transferred to nitrocellulose

membranes. The blots were blocked with 3% casein, and reacted with MAb diluted 1:5000 followed by alkaline phosphatase (AKP)-conjugated goat anti-mouse IgG (DAKO) and then developed with bromochloroindole phosphate/nitro blue tetrazalium substrate (BCIP/NBT).

The enhanced reactivity of 8H3 to rE2 mutants by 8C11

The spatial relationships of epitope recognition by each of the MAbs were investigated by a "Enhancement CLIA" experiment in which 30ng/well rE2 mutant antigen was coated to the microplate, then the solid surface was incubated with saturating levels of unlabeled 8C11 prior to the addition of HRP-conjugated 8H3. The HRP-conjugated MAbs were diluted in PBS containing 20% bovine sera to a concentration that resulted in a final RLU value between 5.0×10^6 and 1.0×10^7 in an indirect CLIA. Samples of unlabeled 8C11 (100 μl, 20 μg/ml in PBST) were added to a rE2-coated microplate for 30min at 37◦C, and aspirated out. Then 100 μl of suitable diluted HRP-conjugated 8H3 were added for a further 30min at 37◦C. The wells were washed, 100μl chemiluminescence substrate was added. Wells were read with a chemiluminescence microwell reader 5 minutes later. The enhencing rate was

calculated as percentage of the increased RLU value of the well with 8C11 compared with the well without 8C11. The control was set as the coated well added 8H3-HRP or 8C11-HRP. NE2 was used as a positive control.

References

Huang YX, Bao YL, Guo SY, Wang Y, Zhou CG, Li YX (2008) Pep-3D-Search: a method for B-cell epitope prediction based on mimotope analysis. BMC bioinformatics 9: 538

Hwang H, Vreven T, Pierce BG, Hung JH, Weng Z (2010) Performance of ZDOCK and ZRANK in CAPRI rounds 13-19. Proteins 78: 3104-3110

Luo WX, Zhang J, Yang HJ, Li SW, Xie XY, Pang SQ, Li SJ, Xia NS (2000) Construction and application of an Escherichia coli high effective expression vector with an enhancer. Sheng wu gong cheng xue bao 16: 578-581

Tsodikov OV, Record MT, Jr, Sergeev YV (2002) Novel computer program for fast

exact calculation of accessible and molecular surface areas and average surface curvature, J Comput Chem 23: 600-609

Vreven T, Pierce BG, Hwang H, Weng Z (2013) Performance of ZDOCK in CAPRI rounds 20-26, Proteins.
